# Supplementary material for: Systemic and Local Medical or Surgical Therapies for Ear, Nose and/or Throat Manifestations in ANCA-Associated Vasculitis: A Systematic Literature Review
Source: J Clin Med. 2023 Apr 28;12(9):3173. doi: 10.3390/jcm12093173 (PMC10179364; doi:10.3390/jcm12093173)
Supplement: Supplementary file 1 [file jcm-12-03173-s001.zip › jcm-2362243-supplementary.pdf]

Supplementary material

Supplementary Material S1: Search string

A search with these search strings was performed on 10-01-2022, applying a filter for publication date from 01-01-2005 till 01-01-2022

Pubmed

(((((((((((((((Otorhinolaryngologic Diseases[MeSH Terms]) OR Ear diseases[MeSH Terms]) OR Laryngeal diseases[MeSH Terms]) OR Nose diseases[MeSH Terms]) OR Otorhinolaryngologic*[Title/Abstract]) OR Otolaryngologic*[Title/Abstract]) OR ENT[Title/Abstract]) OR ear[Title/Abstract]) OR nose[Title/Abstract]) OR throat[Title/Abstract]) OR otologic*[Title/Abstract]) OR laryn*[Title/Abstract]) OR rhinolog*[Title/Abstract]) OR nasal[Title/Abstract])) AND (((((((((((((((((((((Anti-Neutrophil Cytoplasmic Antibody-Associated Vasculitis[MeSH Terms]) OR Churg-Strauss Syndrome[MeSH Terms]) OR Granulomatosis with Polyangiitis[MeSH Terms]) OR Microscopic Polyangiitis[MeSH Terms]) OR Anti Neutrophil Cytoplasmic Antibody Associated Vasculitis[Title/Abstract]) OR Anti-Neutrophil Cytoplasmic Antibody-Associated Vasculitis[Title/Abstract]) OR ANCA-Associated Vasculit*[Title/Abstract]) OR ANCA Associated Vasculit*[Title/Abstract]) OR AAV[Title/Abstract]) OR Pauci*[Title/Abstract]) OR Churg-Strauss[Title/Abstract]) OR Churg Strauss[Title/Abstract]) OR Granulomatous Angiit*[Title/Abstract]) OR Allergic angiit*[Title/Abstract]) OR EGPA[Title/Abstract]) OR Eosinophilic Granulomatosis with Polyangiit*[Title/Abstract]) OR Granulomatosis with Polyangiit*[Title/Abstract]) OR Wegener*[Title/Abstract]) OR GPA[Title/Abstract]) OR MPA[Title/Abstract]) OR Microscopic Polyangiit*[Title/Abstract])

Embase

('ear nose throat disease'/exp OR 'ear disease'/exp OR 'nose disease'/exp OR 'throat disease'/exp OR 'otorhinolaryngologic*':ti,ab,kw OR 'otolaryngologic*':ti,ab,kw OR 'ent':ti,ab,kw OR 'ear':ti,ab,kw OR 'nose':ti,ab,kw OR 'throat':ti,ab,kw OR 'otologic*':ti,ab,kw OR 'laryn*':ti,ab,kw OR 'rhinolog*':ti,ab,kw OR 'nasal*':ti,ab,kw) AND ('anca associated vasculitis'/exp OR 'churg strauss syndrome'/exp OR 'granulomatous vasculitis'/exp OR 'microscopic polyangiitis'/exp OR 'wegener granulomatosis'/exp OR 'anti neutrophil

cytoplasmic antibody associated vasculitis':ti,ab,kw OR 'anti-neutrophil cytoplasmic antibody-associated vasculitis':ti,ab,kw OR 'anca associated vasculit*':ti,ab,kw OR 'anca-associated vasculit*':ti,ab,kw OR 'aav':ti,ab,kw OR 'pauci*':ti,ab,kw OR 'churg-strauss':ti,ab,kw OR 'churg strauss':ti,ab,kw OR 'granulomatous angiit*':ti,ab,kw OR 'egpa':ti,ab,kw OR 'eosinophilic granulomatosis with polyangiit*':ti,ab,kw OR 'granulomatosis with polyangiit*':ti,ab,kw OR 'wegener*':ti,ab,kw OR 'gpa':ti,ab,kw OR 'mpa':ti,ab,kw OR 'microscopic polyangiit*':ti,ab,kw)

Supplementary Table S1. PICO formulated question

**Question:** What is the effect of systemic immunosuppressive treatment or local therapy on ENT activity in patients with AAV and ENT involvement?
Population: Patients with AAV and ENT involvement<br>Intervention: systemic immunosuppressive therapy or local therapy<br>Comparison: different immunosuppressive agents or other local therapies, no systemic immunosuppressive therapy of local therapy, no control group<br>Outcome: ENT disease activity

Supplementary Table S2. Levels of evidence according to the Oxford centre for evidence-based medicine [27]

Level	Description
1a	Systematic review of randomized controlled trials
1b	Individual randomized controlled trial
2a	Systematic review of cohort studies
2b	Individual cohort studies (and low quality randomized controlled trials)
3a	Systematic review of case-control studies
3b	Individual case-control studies
4	Case-series (and poor quality cohort and case-control studies)

Supplementary Table S3. Overview of all articles included in full-text assessment

**Article name**	**First author**
Airway involvement in granulomatosis with polyangiits patients	Acharya
Rituximab for the treatment of eosinophilic granulomatosis with polyangiitis: A systematic literature review	Akiyama
Upper airway manifestations of granulomatosis with polyangiitis.	Alam
Current Therapeutic Approaches to Subglottic Stenosis in Patients With GPA: A Systematic Review	Almuhanna
New treatment of subglottic stenosis due to Wegener's granulomatosis	Arebro
Otologic Manifestations of Eosinophilic Granulomatosis With Polyangiitis: A Systematic Review	Ashman
Ear, nose and throat manifestations of Churg-Strauss syndrome	Bacciu
Nasal Polyposis in Churg-Strauss Syndrome	Bacciu
Anti-cytokine targeted therapies for ANCA-associated vasculitis	Bala
Granulomatosis with polyangiitis in Tunisia	Ben Ghorbel
Staphylococcus Aureus carriage and long-term Rituximab treatment for Granulomatosis with polyangiitis	Besada
Mepolizumab for Eosinophilic Granulomatosis With Polyangiitis: A European Multicenter Observational Study.	Bettiol
Sinonasal Wegener granulomatosis: a single-institution experience with 120 cases.	Cannady
Head and neck manifestations of granulomatosis with polyangiitis: A Retrospective analysis of 19 Patients and Review of the Literature	Carnevale
Presentation, Diagnosis and Management of Subglottic and Tracheal Stenosis During Systemic Inflammatory Diseases	Catano
Rituximab prescription patterns and efficacy in the induction treatment of ANCA-Associated Vasculitis in a Belgian multicenter cohort.	Chasseur
Factors Affecting Dilation Interval in Patients With Granulomatosis With Polyangiitis-Associated Subglottic and Glottic Stenosis	Chen
Clinical [corrected] and pathological characteristics of Chinese patients with antineutrophil cytoplasmic autoantibody associated systemic vasculitides: a study of 426 patients from a single centre.	Chen
Trimethoprim-sulfamethoxazole and antineutrophil cytoplasmic antibodies-associated vasculitis.	Cohen Tervaert
What is the best treatment option for granulomatosis with polyangiitis?	Comarmond
Granulomatosis with polyangiitis (Wegener): clinical aspects and treatment.	Comarmond
Saddle nose deformity and septal perforation in granulomatosis with polyangiitis.	Coordes
Eosinophilic granulomatosis with polyangiitis (Churg-Strauss)	Cordier
The complexity of classifying ANCA-associated small-vessel vasculitis in actual clinical practice: data from a multicenter retrospective survey.	Corral-Gudino

Subglottic Stenosis in Granulomatosis With Polyangiitis: The Role of Laryngotracheal Resection.	Costantino
Features at presentation in a series of patients (n=10) with Churg-Strauss syndrome	Coulter
Randomized trial of cyclophosphamide versus methotrexate for induction of remission in early systemic antineutrophil cytoplasmic antibody-associated vasculitis.	De Groot
Wegener's granulomatosis: experience from a Brazilian tertiary center.	de Souza
Real-life evidence of low-dose mepolizumab efficacy in EGPA: a case series.	Detoraki
Long-Term Followup of a Multicenter Cohort of 101 Patients With Eosinophilic Granulomatosis With Polyangiitis (Churg-Strauss)	Durel
Wegener's granulomatosis: current trends in diagnosis and management.	Erickson
Nine patients with anti-neutrophil cytoplasmic antibody-positive vasculitis successfully treated with rituximab.	Eriksson
Ear, nose and throat involvement in granulomatosis with polyangiitis: how it presents and how it determines disease severity and long-term outcomes	Felicett
Intratracheal Dilation-injection Technique in the Treatment of Granulomatosis with Polyangiitis Patients with Subglottic Stenosis	Fijolek
Disease heterogeneity in antineutrophil cytoplasmic antibody-associated vasculitis: implications for therapeutic approaches	Ford
Granulomatous disorders of the nose and paranasal sinuses.	Fuchs
Rituximab in relapsed/refractory antineutrophil cytoplasmic antibody associated vasculitis: A single-center prospective observational study	Gayatri
Head and neck manifestations of Wegener's granulomatosis.	Gottschlich
Treatment for adult idiopathic and Wegener-associated subglottic stenosis.	Gouveris
Clinic manifestations in granulomatosis with polyangiitis	Greco
Microscopic polyangiitis: Advances in diagnostic and therapeutic approaches	Greco
Multilevel airway stenosis in patients with granulomatosis with polyangiitis (Wegener's).	Guardiani
Benralizumab as a Steroid-Sparing Treatment Option in Eosinophilic Granulomatosis with Polyangiitis	Guntur
Otorhinolaryngological findings in a group of patients with rheumatic diseases.	Gusmao
Clinical features and treatment outcomes of otitis media with antineutrophil cytoplasmic antibody (ANCA)-associated vasculitis (OMAAV): A retrospective analysis of 235 patients from a nationwide survey in Japan.	Harabuchi
Morbidity in patients with ANCA-associated vasculitis	Harper
ANCA-associated vasculitis: diagnosis, clinical characteristics and treatment	Haubitz
Surgical interventions and local therapy for Wegener's granulomatosis.	Hernandez-Rodriguez

Mycophenolate Mofetil vs Azathioprine for Remission Maintenance in Antineutrophil Cytoplasmic Antibody–Associated VasculitisA Randomized Controlled Trial	Hiemstra
[Liposomal local therapy of sinunasal symptoms in ANCA associated vasculitis].	Hofauer
ANCA-associated vasculitides: pathogenetic aspects and current evidence-based therapy.	Holle
Rituximab for refractory granulomatosis with polyangiitis (Wegener's granulomatosis): comparison of efficacy in granulomatous versus vasculitic manifestations.	Holle
Impact of Paranasal Sinus Surgery in Granulomatosis With Polyangiitis: A Longitudinal Computed Tomography Study	Holme
The association between ear involvement and clinical features and prognosis in ANCA-associated vasculitis	Hosokawa
Subglottic stenosis: a ten-year review of treatment outcomes.	Hseu
Localised Versus Systemic Granulomatosis with Polyangiitis: Data from the French Vasculitis Study Group Registry.	Iudici
The hearing prognosis of otitis media with ANCA-associated vasculitis	Iwata
Rituximab for ANCA-associated vasculitis: The UK experience	Jayne
Is rituximab 'The Wonder Drug' for antineutrophil cytoplasmic antibodies-associated vasculitis?	Jha
Systematic review of safety and efficacy of belimumab in treating immune-mediated disorders	Kaegi
What is the evidence for prophylactic antibiotic treatment in patients with systemic vasculitides?	Kallenberg
Oral corticosteroid-sparing effects of reslizumab in the treatment of eosinophilic granulomatosis with polyangiitis	Kent
Inflammatory diseases of the nose and the paranasal sinuses	Klimek
Clinical aspects of granulomatosis with polyangiitis affecting the head and neck.	Knopf
Chapter 11: Granulomatous diseases and chronic sinusitis.	Kohanski
Manifestation of granulomatosis with polyangiitis in head and neck.	Kuhn
Clinical profile and long-term outcome of granulomatosis with polyangiitis (GPA): A corporate hospital-based study from northern India	Kumar
Effectiveness of Rituximab for the Otolaryngologic Manifestations of Granulomatosis With Polyangiitis (Wegener's)	Lally
Update on clinical, pathophysiological and therapeutic aspects in ANCA-associated vasculitides.	Lamprecht
Update on the treatment of granulomatosis with polyangiitis (Wegener's)	Langford
Modern management of primary systemic vasculitis	Lapraik
[Diagnosis, therapy and current research aspects of selected chronic inflammatory diseases with head and neck involvement].	Laudien

Chronic rhinosinusitis in eosinophilic granulomatosis with polyangiitis: clinical presentation and antineutrophil cytoplasmic antibodies	Low
Diagnosis and classification of granulomatosis with polyangiitis (aka Wegener's granulomatosis)	Lutalo
Otolaryngological progression of granulomatosis with polyangiitis after systemic treatment with rituximab.	Malm
Localized Wegener's granulomatosis.	Maranzo
Methotrexate vs cyclophosphamide as maintenance therapy in severe eosinophilic granulomatosis with polyangitiis: a subanalysis of the powercime trial	Maritati
Long-term damage to the ENT system in Wegener's granulomatosis	Martinez del Pero
Off-label use of biologics for the treatment of refractory and/or relapsing granulomatosis with polyangiitis.	Mettler
Efficacy of glucocorticoids to treat limited flares in ancaassociated vasculitis	Miloslavsky
Outcomes of nonsevere relapses in antineutrophil cytoplasmic antibody-associated vasculitis treated with glucocorticoids	Miloslavsky
Clinical outcomes of remission induction therapy for severe antineutrophil cytoplasmic antibody-associated vasculitis	Miloslavsky
Vestibular Involvement in Patients With Otitis Media With Antineutrophil Cytoplasmic Antibody-associated Vasculitis.	Morita
Results of endoscopic surgery and intralesional steroid therapy for airway compromise due to tracheobronchial Wegener's granulomatosis.	Nouraei
Successful use of rituximab in six patients with relapsing eosinophilic granulomatosis with polyangiitis	Novikov
The treatment outcomes of rituximab for intractable otitis media with ANCA-associated vasculitis.	Okada
Utility of a one-stop joint ent-vasculitis clinic in the management of relapsing ANCA-associated vasculitis	O'malley
Clinical features and outcomes of 37 Argentinean patients with severe granulomatosis with polyangiitis (wegener granulomatosis).	Orden
Relapses in patients with anti-neutrophil cytoplasmic antibody-associated vasculitis: a retrospective study.	Outh
Azathioprine or methotrexate maintenance for ANCA-associated vasculitis	Pagnoux
Treatment of systemic necrotizing vasculitides in patients aged sixty-five years or older: results of a multicenter, open-label, randomized controlled trial of corticosteroid and cyclophosphamide-based induction therapy	Pagnoux
Wegener's granulomatosis strictly and persistently localized to one organ is rare: assessment of 16 patients from the French Vasculitis Study Group database.	Pagnoux
Office-based corticosteroid injections as adjuvant therapy for subglottic stenosis	Pan
The role of surgery in antineutrophil cytoplasmic antibody-associated vasculitides affecting the nose and sinuses: A systematic review.	Pendolino

Sirolimus use in patients with subglottic stenosis in the context of granulomatosis with polyangiitis (GPA), suspected GPA, and immunoglobulin G4-related disease	Poo
Non-severe eosinophilic granulomatosis with polyangiitis: long-term outcomes after remission-induction trial.	Puechal
Adding Azathioprine to Remission-Induction Glucocorticoids for Eosinophilic Granulomatosis With Polyangiitis (Churg-Strauss), Microscopic Polyangiitis, or Polyarteritis Nodosa Without Poor Prognosis Factors: A Randomized, Controlled Trial.	Puechal
Treatment of Polyarteritis Nodosa and Microscopic Polyangiitis Without Poor-Prognosis Factors	Ribi
Treatment of Churg-Strauss syndrome without poor-prognosis factors: a multicenter, prospective, randomized, open-label study of seventy-two patients	Ribi
Response to mepolizumab according to disease manifestations in patients with eosinophilic granulomatosis with polyangiitis	Rios-Garces
Damage in the anca-associated vasculitides: Long-term data from the European Vasculitis Study group (EUVAS) therapeutic trials	Robson
Laryngeal and tracheobronchial involvement in Wegener's granulomatosis.	Rodrigues
A Case Series of Granulomatosis With Polyangiitis Primarily Diagnosed by Otological Manifestations.	Sahyouni
Long-term outcomes of 118 patients with eosinophilic granulomatosis with polyangiitis (Churg-Strauss syndrome) enrolled in two prospective trials.	Samson
Dilatation tracheoscopy for laryngeal and tracheal stenosis in patients with Wegener's granulomatosis.	Schokkenbroek
Granulomatosis with polyangiitis: Experience of 42 patients from a single-center, tertiary care hospital in Mumbai	Singh
Granulomatosis with polyangiits: Clinical manifestations, laboratory parameters, and outcome of 26 patients from a single center in Mumbai, Maharashtra, India	Singh
Clinical features and therapeutic management of subglottic stenosis in patients with Wegener's granulomatosis.	Solans-Laque
Clinical characteristics and outcome of Spanish patients with ANCA-associated vasculitides: Impact of the vasculitis type, ANCA specificity, and treatment on mortality and morbidity.	Solans-Laque
Rituximab plus methotrexate combination as a salvage therapy in persistently active granulomatosis with polyangiitis	Sorin
Patterns of presentation and diagnosis of patients with Wegener's granulomatosis: ENT aspects.	Srouji
Evaluation of clinical benefit from treatment with mepolizumab for patients with eosinophilic granulomatosis with polyangiitis	Steinfield
Technique of laryngotracheal resection in subglottic stenosis.	Stoelben

Rituximab versus cyclophosphamide for induction of remission in ANCA-associated vasculitis: a randomized controlled trial (RAVE)	Stone
Wegener's granulomatosis causing subglottic stenosis: Experiences at a tertiary care hospital of the Eastern India	Swain
Otitis media with ANCA-associated vasculitis: A retrospective study of 30 patients.	Tabei
Survival of patients with Wegener's granulomatosis diagnosed in Finland in 1981-2000.	Takala
No evident association of nasal carriage of Staphylococcus aureus or its small-colony variants with cotrimoxazole use or ANCA-associated vasculitis relapses.	Tan
Clinical manifestations and treatment of idiopathic and Wegener granulomatosis-associated subglottic stenosis.	Taylor
Progression and management of Wegener's granulomatosis in the head and neck.	Taylor
Efficacy and safety of rituximab in the treatment of eosinophilic granulomatosis with polyangiitis	Teixeira
Clinical Features of Patients with Active Eosinophilic Granulomatosis with Polyangiitis Successfully Treated with Mepolizumab	Tsurikisawa
Effectiveness and safety of mepolizumab in combination with corticosteroids in patients with eosinophilic granulomatosis with polyangiitis	Ueno
Optimizing the therapeutic strategies in ANCA-associated vasculitis--single centre experience with international randomized trials.	Vanková
Mepolizumab or Placebo for Eosinophilic Granulomatosis with Polyangiitis.	Wechsler
Effect of Treatment on Damage and Hospitalization in Elderly Patients with Microscopic Polyangiitis and Granulomatosis with Polyangiitis	Weiner
Otologic symptoms as initial manifestation of wegener granulomatosis: diagnostic dilemma.	Wierzbicka
Intralesional corticosteroid injection and dilatation provides effective management of subglottic stenosis in Wegener's granulomatosis.	Wolter
Management of anca-associated vasculitis in the UK: A complex pathway of patient referral, diagnosis, and treatment	Worthington
Clinical and serological features of eosinophilic and vasculitic phases of eosinophilic granulomatosis with poliangiitis: A case series of 15 patients	Yilmaz
Reversible cochlear function with ANCA-associated vasculitis initially diagnosed by otologic symptoms.	Yoshida
The Liverpool experience: The role of immunosuppression in treating vasculitic subglottic stenosis	Zammit
Subglottic and tracheal stenosis due to Wegener's granulomatosis.	Zycinska
Co-trimoxazole and prevention of relapses of PR3-ANCA positive vasculitis with pulmonary involvement.	Zycinska
